# Draft Genome Sequence of *Cytophaga fermentans* JCM 21142^T^, a Facultative Anaerobe Isolated from Marine Mud

**DOI:** 10.1128/genomeA.00206-14

**Published:** 2014-03-27

**Authors:** David Starns, Kenshiro Oshima, Wataru Suda, Takao Iino, Masahiro Yuki, Jun-Ichi Inoue, Keiko Kitamura, Toshiya Iida, Alistair Darby, Masahira Hattori, Moriya Ohkuma

**Affiliations:** a Japan Collection of Microorganisms/Microbe Division, RIKEN BioResource Center, Tsukuba, Ibaraki, Japan; b Institute of Integrative Biology, University of Liverpool, Liverpool, United Kingdom; c Center for Omics and Bioinformatics, Graduate School of Frontier Sciences, the University of Tokyo, Kashiwa, Chiba, Japan; d Biomass Research Platform Team, RIKEN Biomass Engineering Program Cooperation Division, RIKEN Center for Sustainable Resource Science, Tsukuba, Ibaraki, Japan

## Abstract

*Cytophaga fermentans* strain JCM 21142^T^ is a marine-dwelling facultative anaerobe. The draft genome sequence of this strain revealed its diverse chemoorganotrophic potential, which makes it capable of metabolizing various polysaccharide substrates. The genome data will facilitate further studies on its taxonomic reclassification, its metabolism, and the mechanisms pertaining to bacterial gliding.

## GENOME ANNOUNCEMENT

The bacterium *Cytophaga fermentans*, belonging to the phylum *Bacteriodetes* and first described in 1955 (1), is found commonly in marine mud, near shores, and on decaying marine organisms (2). Metabolic studies on the species in the order *Cytophagales* have shown that they are diverse chemoorganotrophs able to degrade many biomacromolecular compounds, including chitin and cellulose (2). *C. fermentans* is a rod-shaped, Gram-negative, facultative anaerobe, with anaerobic growth at the expense of organic compounds (1). Like other cytophagas, it is motile, using gliding to move across solid surfaces. The mechanism for gliding still remains to be clarified but recently a model was created for *Flavobacterium johnsoniae* in which helical surface proteins are thought to play a crucial role (3). Although gliding motility is a typical feature of cytophagas, phylogenetic analyses based on the 16S rRNA gene sequence revealed that *C. fermentans* is misclassified as a species of *Cytophaga* and should be classified to the order *Bacteroidales*, not to *Cytophagales* (4, 5).

The type strain *Cytophaga fermentans* JCM 21142 was sequenced *de novo* using the Ion Torrent PGM system, generating 1,115,426 quality-filtered reads. These reads were assembled using Newbler version 2.8 (Roche) into 299 contigs (longest, 218,358 bp) with an *N*_50_ length of 59,017 bp. The resulting draft genome sequence is 5,649,512 bp, with 38.6× redundancy and a G+C content of 37.4%. The draft genome sequence was annotated using the Victoria Bioinformatics Consortiums’ pipeline Prokka (Prokaryotic Genome Annotation System) version 1.5.2 and the genome annotation was completed using Prodigal (6) version 2.6, to identify 4,781 protein coding sequences. Aragorn (7) version 1.2.34 predicted 46 tRNAs, Infernal (8) version 1.1 predicted 24 noncoding RNAs (ncRNAs), and RNAmmer (9) version 1.2. predicted 3 rRNAs.

From the annotation, 580 genes and gene fragments were assigned to 78 different Carbohydrate-Active Enzyme database (CAZy) families (10). Those identified included genes for galactosidases, mannosidases, xylanases, chitinases, glucosidases, glucanases, and agarases, suggesting the utilization of various compounds as carbon and energy sources. The annotation also uncovered genes for carbon fixation, supporting the CO_2_-requiring nature of *C. fermentans* in a defined medium (1), as well as bacterial gliding membrane protein families Gld and Spr, which could allow further elucidation of the mechanism of bacterial gliding. The sequence data and annotated genome will allow improved comparative phylogenetic analyses to better establish the *C. fermentans* taxonomic position within the *Bacteriodetes* phylum.

### Nucleotide sequence accession numbers.

The genome sequence of *Cytophaga fermentans* strain JCM 21142^T^ has been deposited in DDBJ/EMBL/Genbank under the accession numbers BAMD01000001 through BAMD01000299.
